# Single-center, prospective phase 2 trial of high-intensity focused ultrasound (HIFU) in patients with unilateral localized prostate cancer: good functional results but oncologically not as safe as expected

**DOI:** 10.1007/s00345-023-04352-9

**Published:** 2023-03-15

**Authors:** Gregor Duwe, Katharina Boehm, Maximilian Haack, Peter Sparwasser, Maximilian Peter Brandt, Rene Mager, Igor Tsaur, Axel Haferkamp, Thomas Höfner

**Affiliations:** 1 Department of Urology and Pediatric Urology, University Medical Center Johannes-Gutenberg University, Langenbeckstraße 1, 55131 Mainz, Germany; 2 Department of Urology, Carl-Gustav-Carus University Medical Center, Dresden, Germany; 3 Department of Urology, Ordensklinikum Linz Elisabethinen, Linz, Austria

**Keywords:** Prostate cancer, Unilateral localized prostate cancer, Focal therapy, High intensity focused ultrasound, HIFU

## Abstract

**Purpose:**

Focal therapy (FT) for localized prostate cancer (PCa) is only recommended within the context of clinical trials by international guidelines. We aimed to investigate oncological follow-up and safety data of focal high-intensity focused ultrasound (HIFU) treatment.

**Methods:**

We conducted a single-center prospective study of 29 patients with PCa treated with (focal) HIFU between 2016 and 2021. Inclusion criteria were unilateral PCa detected by mpMRI-US-fusion prostate biopsy and maximum prostate specific antigen (PSA) of 15 ng/ml. Follow-up included mpMRI-US fusion-re-biopsies 12 and 24 months after HIFU. No re-treatment of HIFU was allowed. The primary endpoint was failure-free survival (FFS), defined as freedom from intervention due to cancer progression.

**Results:**

Median follow-up of all patients was 23 months, median age was 67 years and median preoperative PSA was 6.8 ng/ml. One year after HIFU treatment PCa was still detected in 13/ 29 patients histologically (44.8%). Two years after HIFU another 7/29 patients (24.1%) were diagnosed with PCa. Until now, PCa recurrence was detected in 11/29 patients (37.93%) which represents an FFS rate of 62%.One patient developed local metastatic disease 2 years after focal HIFU. Adverse events (AE) were low with 70% of patients remaining with sufficient erectile function for intercourse and 97% reporting full maintenance of urinary continence.

**Conclusion:**

HIFU treatment in carefully selected patients is feasible. However, HIFU was oncologically not as safe as expected because of progression rates of 37.93% and risk of progression towards metastatic disease. Thus, we stopped usage of HIFU in our department.

**Supplementary Information:**

The online version contains supplementary material available at 10.1007/s00345-023-04352-9.

## Introduction

Focal therapy (FT) for prostate cancer (PCa) gained large interest by urologists and patients in treatment of low and intermediate-risk disease with the aim to provide equivalent oncological safety while reducing adverse events (AE) due to radical treatment [1]. The increased interest for FT can be explained by the growing number of younger patients who are identified at an earlier stage with localized PCa and small-volume prostate lesions due to the successful screening programs [2].

Most FT are based on ablative technologies, in particular high-intensity focused ultrasound (HIFU), cryotherapy, photodynamic therapy, electroporation, and focal radiation therapy RT by brachytherapy, for example [3]. In brief, HIFU is the most widely used technology with highest number of clinical retrospective data available. HIFU technology uses a transducer that ablates prostate tissue up to 65 degrees Celsius (149 degrees Fahrenheit), destroying malignant tumor cells through shear forces [4].

Currently, FTs are still defined in oncological guidelines as experimental treatment options that should only be performed in the context of clinical trials due to the significant lack of robust prospective clinical trials [5]. Nevertheless, large surveys in western urological communities’ report that every second urologist in Europe and one of four urologists in the United States recommend and perform FT, even outside clinical trials [6].

The main argument for HIFU is based on the index lesion theory which states that there is a pre-dominant focus of PCa causing local and systemic progression of the disease [7]. Consequently, FT is selectively treating this primary tumor focus while protecting presumably non-affected prostate tissue and leaving insignificant foci under surveillance. Pretherapeutic multiparametric MRI (mpMRI) and mpMRI-targeted biopsy represents the fundamental diagnostic technique pre-selecting patients and monitoring treatment outcomes. Multiple studies have reported high sensitivity (up to 90%) for mpMRI-targeted in combination with systematic biopsies in detecting significant PCa lesions [8]. Consequently, there is a remaining diagnostic uncertainty of mpMRI between 9 and 11% to miss significant PCa (ISUP grade ≥ 2) [9, 10]. Even though PCa is considered a multifocal disease, various studies reported a limited involvement to a singular lesion in 13–38%, and unilateral involvement in 19–63% [11, 12].

We aimed to assess the clinical outcome of patients treated by FT using HIFU in a prospective trial using rigorous inclusion and follow-up criteria. Based on previous HIFU data, we expected to reduce the risk of disease progression using HIFU only in carefully selected patients while reducing AE compared to standard recommended therapies like RP and RT.

## Patients and methods

This controlled, prospective, non-randomized, phase 2 clinical study was carried out in a single European institution between December 2016 and October 2021. Follow-up is still ongoing. All patients gave written informed consent and were extensively informed about established treatment recommendations of RP or RT. The local ethical board approved the study (2019-14595). All patients received mpMRI of the prostate, followed by mpMRI-ultrasound (US)-fusion prostate biopsy of suspicious lesions with a score of ≥ 3 on the Prostate Imaging-Reporting and Data System (PI-RADS)*.* Additional 12 cores were taken for a systematic examination. Only patients who had been diagnosed with unilateral PCa of ISUP grade 1 to 3 were included in the study and treated with focal HIFU*.* Further inclusion criteria involved prostate specific antigen (PSA) ≤ 15 ng/ml, clinical tumor classification ≤ T2 and prostate volume of ≤ 90 ml due to the technical limitation in insufficiently reaching ventral gland areas with the rectal HIFU transducer. Afterwards all patients received HIFU Focal One ^®^ (EDAP TMS SA) therapy.

Follow-up protocol included PSA blood tests every three months during the first 2 years after HIFU treatment. At first and second year after HIFU treatment mpMRI and MRI-US fusion biopsies were conducted. AE after treatment were documented, using standard Clavien-Dindo classification. Pre-therapeutic diagnostic procedures, HIFU treatment and follow-up protocols were conducted at the same center. As part of the study protocol, we set up a goal of maximum 20% progression to more aggressive treatments, as this is a reasonable goal to achieve benefits compared to progression risk of Active Surveillance (AS) and based on recommendations by expert consensus statements to define a good focal therapy center [13]. The study protocol was based on the international recommendation valid at that time. Thus, the following PSA levels after HIFU treatment were individually evaluated in our multidisciplinary tumor board before performing re-biopsy to define treatment failure, as there was no established threshold for the PSA level defining treatment failure: rise of PSA above patient baseline PSA value and significant increase of PSA as compared to PSA nadir [13, 14]. If the PSA level increased suspiciously, mpMRI-targeted biopsy was recommended. Finally, metastatic progression during follow-up was defined as criteria to stop the trial.

As a unique exception from other centers, we strictly excluded focal HIFU retreatments in our protocol [15]. We also excluded patients with any previous therapy for PCa (AS, RT, hormone therapy), patients without prostate-limited disease preoperatively (in terms of extracapsular extension, seminal vesicle invasion or lymph nodes metastases), as well as patients diagnosed with PCa after transurethral resection of the prostate.

First, pre-interventional patient characteristics such as age at intervention, PSA level, prostate volume, HIFU therapy (hemi-ablation vs. focal ablation), PI-RADS classification, Gleason grading, number of positive cores in the targeted biopsy cores and number of positive cores in the systematic biopsy cores were described and stratified according to cancer recurrence at follow-up. Failure free survival (FFS), defined as freedom from any further intervention (e.g., RP or RT) due to cancer progression, was the primary outcome of the study. Secondary outcomes included total PSA levels, PSA nadir, area of recurrence diagnosed in mpMRI as part of follow-up and AE.

Statistical analysis was performed using IBM SPSS Statistics Version 27 (Armonk, NY: IBM Corp.). Descriptive statistics were reported as frequencies and proportions, continuous variables were presented as medians ± interquartile range (IQR, 25–75). Continuous variables were compared using the Mann–Whitney U test. FFS was demonstrated by Kaplan–Meier curves.

## Results

Baseline characteristics of the study population are summarized in Table 1. Overall, a total of 29 men were enrolled in this study. Follow-up and AS are still ongoing. Median follow-up was 23 months and median age at time of focal HIFU treatment was 67 years. Median time to reach PSA nadir was six months.

**Table 1:** Baseline patient characteristics and outcome parameter

Variable	Number of patients (*n = *29)
Patient characterictics	Median (IQR)
Age (years)	66.0 (61.5;72.5)
Prostata volume (ml)	42.0 (29.5;57.0)
iPSA (ng/ml)	6.8 (5.1;8.9)
(Pre-) treatment parameters (mpMRI, *n = *27)	Total number (%)
No PIRDAS lesion	2 (7.4%)
PIRDAS-2	2 (7.4%)
PIRDAS-3	4 (14.8%)
PIRDAS-4	18 (66.6%)
PIRDAS-5	1 (3.7%)
ISUP grade 1 (Gleason Score 3 + 3 = 6)	20 (69%)
ISUP grade 2 (Gleason Score 3 + 4 = 7a)	8 (27.6%)
ISUP grade 3 (Gleason Score 4 + 3 = 7b)	1 (3.45%)
HIFU focal ablation	11 (37.9%)
HIFU hemi-ablation	18 (62.1%)
PSA levels (ng/ml)	Median (IQR)
PSA 3 months after HIFU (ng/ml), *n = *26	2.2 (0.99;4.04)
PSA 6 months after HIFU (ng/ml), *n = *27	1.8 (0.91;3.32)
PSA 12 months after HIFU (ng/ml), *n = *20	2.35 (0.85;4.18)
PSA 18 months after HIFU (ng/ml), *n = *15	2.79 (1.47;4.6)
Pathology results 1 year after HIFU treatment	Total number (*n*)
No re-biopsy yet	5 (17.2%)
No malignancy	11 (37.9%)
ISUP grade 1 (Gleason Score 3 + 3 = 6)	11 (37.9%)
ISUP grade 2 (Gleason Score 3 + 4 = 7a)	2 (6.9%)
ISUP grade 3 (Gleason Score 4 + 3 = 7b)	0
Pathology results 2 years after HIFU treatment	Total Number (*n*)
No 2nd re-biopsy yet	17 (58.6%)
No malignancy	5 (17.2%)
ISUP grade 1 (Gleason Score 3 + 3 = 6)	5 (17.2%)
ISUP grade 2 (Gleason Score 3 + 4 = 7a)	2 (6.9%)
ISUP grade 3 (Gleason Score 4 + 3 = 7b)	0
Failure free survival	Total number (*n*)
Recommendation for radical prostatectomy 1 year after HIFU	5 (17.2%)
Recommendation for radical prostatectomy 2 years after HIFU	6 (20.7%)
Months until treatment failure/ drop out (median, IQR)	22.0 (11.0;26.0)
Adverse Events (Clavien-Dindo classification), *n = *29	Total number (*n*)
Haematuria (I°)	7 (24%)
Intense urge to urinate, temporarily, no incontinence (I°)	2 (7%)
Urge incontinence grad 1 (I°)	2 (7%)
Erectile Dysfunction, usage of PDE-5^a^ inhibitors (II°)	9 (31%)
Lower urinary tract infection, epididymitis (II°)	4 (14%)
Urinary retention, temporarily (II°)	5 (17%)
Subvesical obstruction, LUTS^b^, deobstruction recommended (III°)	4 (14%)
No adverse events	4 (14%)

^a^Phosphodiesterase 5 inhibitors

^b^Lower urinary tract symptoms

At first-year follow-up, out of 24 patients who underwent first re-biopsy 14 patients (44.8%) were diagnosed with PCa. Five of these patients (17.2% related to overall study population) were recommended RP or RT because of progression and unsuitability for continuing AS strategy (Table 1) [16]. At 2-year follow-up 12 patients received targeted prostate biopsy with 12-fold systematic biopsy in case of mpMRI lesions. Seven patients out of 12 (24.1%) were diagnosed with PCa. Six of these 12 patients (20.7% of overall population) were recommended RP or RT because of progression. Among all patients with PCa recurrence 2 years after HIFU treatment, six patients (40%) were detected with outfield recurrence regarding initially treated PCa lesions, while five patients (33.33%) showed infield and outfield lesions. Thus, 11 patients (73.33%) developed outfield PCa lesions in our study cohort (Fig. 1 of the Supplementary Material shows a workflow of patients’ follow-up).

**Fig. 1:**
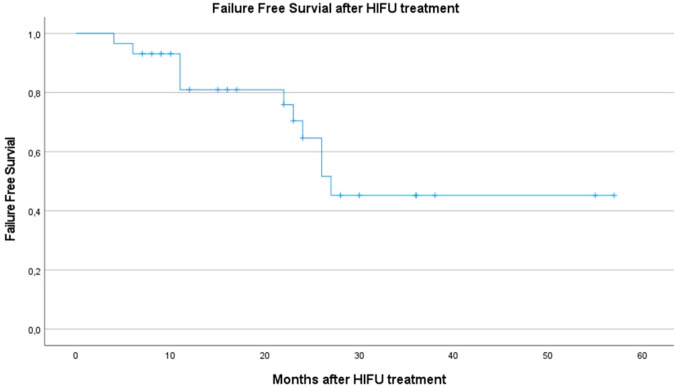
Failure-free survival curve after HIFU treatment

In summary, 2 years after HIFU, we recommended RT or RP treatment in 11 cases which represents a drop-out rate of 37.93% and an FFS rate of 62%, respectively (Fig. 1). Median time until study drop-out was 22 months (standard deviation of 8.77, min. 4 months, max. 27 months).

Notably, we report about one patient who developed lymphatic metastases after focal HIFU treatment. Baseline characteristics of this patient were the following: iPSA of 9.3 ng/ml, prostate volume of 42 ml and ISUP grade 2 unilateral PCa. Initial mpMRI revealed inconspicuous lymph nodes. He underwent HIFU hemi-ablation in March 2020. After initial mpMRI-US fusion biopsy, we performed follow-up biopsy in March 2021 according to study protocol. Here, we detected only one positive biopsy with ISUP grade 1 (< 5% volume of biopsy), thus continued with AS according to guidelines. After reaching low PSA 0.84 ng/ ml nadir, PSA level eventually rose to 6 ng/ml 18 months after HIFU. Two-year follow-up mpMRI demonstrated conspicuous pelvic lymph nodes. Thus, he underwent immediate DaVinci-assisted RP with pelvic lymph node dissection in April 2022. Final pathology revealed pT3 pN1 (6 positive lymph nodes) status. Additional PSMA-PET CT*-*scan confirmed even more cancerous pelvic nodes. Consequently, multimodal adjuvant treatment was recommended with RT plus androgen deprivation therapy.

As a result of the high clinical failure rate of HIFU treatment despite current standard diagnostic methods, with one patient even progressing to metastatic palliative disease, we stopped this clinical study for HIFU treatment in our department because of oncologic safety concerns.

Finally, the most frequent AE, namely gross haematuria, erectile dysfunction, acute urinary retention, urinary tract infection, and urge incontinence are reported in Table 1. Grade 3 Clavien–Dindo complications occurred in 14% of patients. Four patients (14%) had no AE at all. Notably, after 4 years 69% of patients still had full erectile function and 97% reported no effects on their continence.

## Discussion

In our prospective, single-center clinical study of HIFU treatment in unilateral localized PCa we demonstrate high progression rates with overall FFS of only 62%. Despite lower AE compared to RP and RT we had to stop HIFU treatment in localized unilateral PCa because of oncologic safety concerns.

HIFU treatment is based on index lesion theory which has not been properly confirmed, yet. As long as several large pathological analyses of patients undergoing RP reveal multifocal disease in up to 80%, we have to address this knowledge to our patients treated by FT [17]. In our study, 73.33% of patients treated with HIFU revealed outfield PCa lesions in first- or second-year re-biopsies. These findings contradict the index lesion theory and are consistent with larger pathological analyses which support the existing consensus of PCa as multifocal disease [11, 12].

Moreover, high accuracy in optimal patient selection is extremely important for any FT to detect focal disease based on index lesion theory, which is primarily based on mpMRI-US fusion biopsy*.* However, the accuracy of mpMRI in defining clinically significant PCa depends on several variables, as well as interobserver and interoperator variability, which may result in substantial reduction in negative predictive value [8].

Despite our efforts in maximal accurate patient selection to address the underlying index theory of FT, we report an unexpected high number of tumor regression with median FFS rate of approximately 62% of total study cohort (median time until study drop-out of 22 months). Most importantly, one patient developed metastatic disease leading to palliative care. Comparing our results with those of other centers, only few midterm follow-up results are published reporting significant treatment failure and need for re-treatment. One of the largest cohorts has been published in 2019 with a total of 1032 men treated with HIFU between 2005 and 2017 with median follow-up of 36 months [18]. In this study, only 46% of men were free from any need for re-treatment during median follow-up. Approximately 70% of patients receiving any kind of re-treatment underwent a second FT. When regarding men undergoing at least one follow-up biopsy, only 35% were free from clinically significant PCa at 96 months. Another evaluation from 13 centers with 1379 patients and overall median follow-up of 32 months reported 7-years failure-free survival of 69% (64–74%) [19].

Notably, we did not include second HIFU treatment in our study protocol because it prevents causal relationships of cause and effect. Second, we regard high rates of re-treatment by 70% as a critical issue that needs to be discussed, especially when 5-year progression-free survival rates for RP above 80% are documented in large prospective trials [20, 21]. In our study population 37.93% of patients were recommended RP or RT due to tumor progression. Also, progression occurred in initial HIFU treatment areas as well as new areas of the prostate gland which we regard as significant risk to patients’ safety. Moreover, there is no clear consensus on PSA thresholds for acceptable rates of progression under FT [22].

Regarding further smaller study cohorts, PCa recurrence and progression rates after FT treatment also varied from 20 to 30% [23]. These numbers may also reflect the heterogenous and partially unclear inclusion and treatment criteria [24].

Ultimately, our results strengthen the need to increase diagnostic accuracy to detect localized PCa. This could be achieved by further narrowing the inclusion criteria in terms of more localized, small-volume PCa. We propose to establish HIFU study protocols including PSMA PET-CT. Based on the phase III “proPSMA” trial, there is strong evidence for significant improvement in identifying pelvic nodal and distant metastatic PCa compared to conventional CT and bone scanning [25]. When combined with mpMRI-US-fusion prostate biopsy, PSMA PET-CT may lead to improved diagnostic accuracy, thereby reducing outfield recurrences when FT is used.

Nevertheless, we can confirm overall low rates of AE as compared RP or RT [18, 26]. Approximately 70% of our patients maintained full erectile function and 97% reported maintenance of urinary continence while 14% reported no AE at all.

Additionally, we need to address AS treatment for patients with low-risk PCa. Compared to definitive treatment or focal HIFU, AS does not have Clavien ≥ 3 AEs and provides high evidence-based long-term follow-up data showing comparable overall survival in appropriately selected patients. Disease progression rates of AS leading to definitive treatment might also be high, up to approximately 60% in long-term follow-up of 10 years and median FFS up to 8.5 years [27, 28].

Finally, we encourage technical improvements of FT to further increase the effectiveness of ablative technologies to achieve even better local destruction of PCa cells, while better protecting the surrounding tissue, including prostate nerve bundle preservation. Moreover, the usage of HIFU is limited in treatment of prostates larger than 40 ccm or to lesions within 4 cm from the treatment site [29].

We are aware of the limitations of our study, in particular the limited sample size. However, we had to stop the trial after treating 29 patients based on our trial protocol. Thus, we are not able to perform an in-depth multiple logistic regression analysis to identify, for example, risk factors for failure after HIFU treatment. To further evaluate these factors, we would need at least 40–50 patients per parameter. Furthermore, mpMRI-US-fused prostate biopsies were conducted by several urologists and MRI was analyzed by different radiologists, thereby interobserver bias is of specific relevance. Nevertheless, our results resemble real world experience which we believe is very important for colleagues in urology and their daily treatment recommendations.

## Conclusion

In conclusion, FT in PCa using HIFU demonstrated good functional results in patients. However, HIFU was not as safe oncologically as expected, with recurrence and progression rates of 37.93% and risk of disease progression to metastatic disease. We strongly support the further scientific evaluation of FT, but until oncological risks cannot be safely reduced, we no longer recommend HIFU treatment in our department at this time.

## Supplementary Information

Below is the link to the electronic supplementary material.

Supplementary file1 Figure 1: Workflow of 29 patients who received HIFU therapy in our institution. All of them harboured prostate cancer; 20 patients were diagnosed with ISUP 1, eight patients with ISUP 2 and one patient with ISUP 3 prostate cancer. (DOCX 102 kb)
