# Supplementary figures and images for: Single-center, prospective phase 2 trial of high-intensity focused ultrasound (HIFU) in patients with unilateral localized prostate cancer: good functional results but oncologically not as safe as expected
Source: World J Urol. 2023 Mar 15;41(5):1293–9. doi: 10.1007/s00345-023-04352-9 (PMC10188406; doi:10.1007/s00345-023-04352-9)

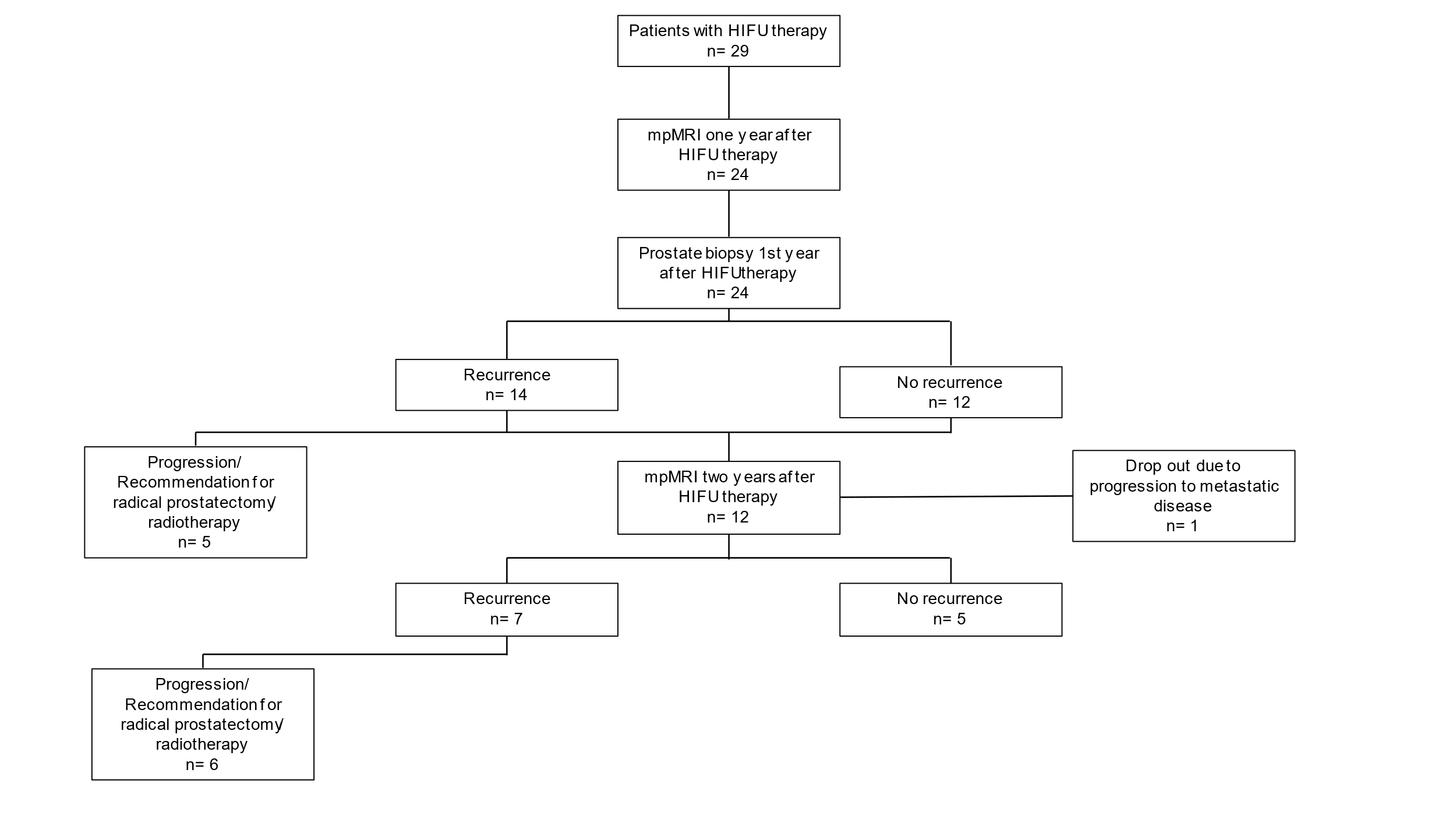

Supplement: Supplementary file 1 — Supplementary file1 Figure 1: Workflow of 29 patients who received HIFU therapy in our institution. All of them harboured prostate cancer; 20 patients were diagnosed with ISUP 1, eight patients with ISUP 2 and one patient with ISUP 3 prostate cancer. (DOCX 102 kb) [file 345_2023_4352_MOESM1_ESM.docx]
